# Disaggregated data to improve child health outcomes

**DOI:** 10.4102/phcfm.v8i1.1221

**Published:** 2016-12-02

**Authors:** Ann M.E.T. Tshabalala, Myra Taylor

**Affiliations:** 1 KwaZulu-Natal Department of Health, Amajuba Health District, South Africa; 2 School of Nursing and Public Health, Discipline of Public Medicine, University of KwaZulu–Natal, South Africa

## Abstract

**Background:**

The District Health Information System was developed in South Africa to collect aggregated routine data from public health facilities. In Amajuba District, KwaZulu-Natal, ward-based data collection has been initiated to facilitate improved responsiveness to community health needs and improve health outcomes and patient satisfaction.

**Aim:**

To assess the application of the municipal ward-based health data in the decision-making process to improve child health outcomes.

**Setting:**

The study was conducted in 25 primary health care service sites in Amajuba.

**Methods:**

A cross-sectional mixed methods’ approach was used. The study population comprised operational managers, professional nurses, ward-based outreach team leaders and supervisors. Quantitative data were collected using a semi-structured questionnaire and analysed using descriptive statistics. Qualitative data were collected using focus group discussions and analysed using thematic analysis.

**Results:**

Of the 131 respondents, 83 (67.5%) provided targeted child interventions to a certain or a large extent to improve child health outcomes, but only 74 (57.4%) respondents reported using municipal ward-based health data to a certain or large extent in order to inform their decisions. This discrepancy indicates poor utilisation of local health information for decision-making.

**Conclusion:**

The study showed that municipal ward-based health data are not fully utilised for making informed decisions to improve child health outcomes. It is imperative to inculcate a culture of evidence-informed decisions that leads to provision of targeted interventions in order to mitigate the challenge of scarcity of resources and to improve child health outcomes.

## Introduction

The District Health Information System (DHIS) was developed to collect routine aggregated data from all public health facilities and is intended to support decentralised decision-making and health service management. Yet, in order to be responsive to the needs of the community there is a need for disaggregated data to enable decision-making at a local level. Understanding the context enhances understanding of population health, who gets sick, from what, and where do they reside.^1,2,3^ Thus, there is a need for collection of municipal ward-based health data and using this to inform the decision-making process in order to increase responsiveness to children’s needs. In South Africa, provinces are divided into districts and sub-districts which are further subdivided into wards. Amajuba district comprises three sub-districts and 46 wards. The municipal ward-based health data collection entails collection of data and documenting the municipal ward to determine the prevalence of different health conditions in each ward. The aim is to disaggregate the data in order to understand the context and increase the responsiveness to actual community needs. The health care workers are expected to document the municipal ward of every patient to whom they attend. This requires collecting the patient’s data, collating, analysing and converting this information for decision-making in the facility. Every month the data are collated and the wards with patients with various health problems are assessed. A summary sheet is completed monthly on the disease burden of the facility’s catchment area. The data are submitted monthly to the district office, and the district office provides feedback quarterly to the facilities on patients from their catchment area that accessed health services elsewhere. This study will contribute to health information transformation in the district. It will enable timely detection and localisation of emerging health problems. Furthermore, it will strengthen the evidence base for effective health policies and contribute to the improvement in the quality of health services and responsiveness to community needs. In view of scarcity of resources in the health sector, it will enhance responsiveness through targeted interventions. The study has identified the need to adequately integrate data in decision-making in order to improve health outcomes and to ensure social and financial risk protection. The findings of the study are aligned to the National Health Insurance (NHI) deliverables of responsiveness to community needs and universal access.

The World Health Organization (WHO) states three ultimate goals of a health system: consumer protection, improved health and financial risk protection.^4^ Other scholars agree with the latter two.^1^ The context within which the system’s function needs to be considered to determine the long- and short-term opportunities and threats faced by the health system. Thus, it is essential to analyse the varied contexts (demographic, economic, political, legal and regulatory, epidemiological, socio-demographic and technological) within which the health systems function.^1^ This is essential for scaling up new programmes and achieving sustained success. Furthermore, to enhance the effectiveness of interventions it is important to consider the local epidemiological profile and the health system characteristics when making decisions in order to ensure responsiveness to community needs.^2,5^ The use of disaggregated information to inform decisions should lead to improved responsiveness to community health needs, improvement of health outcomes and patient satisfaction. This would make the NHI both responsive and accountable, thereby contributing to public value as it is likely to improve user experience, lead to improved health outcomes and a better quality of life.^6^ Improving child health outcomes is of critical concern and child mortality is one of the health outcomes which can be used to assess the functioning of a health system.^2^

In practice, decision-making in health is often based on political considerations and pronouncements, expediency or donor demand, yet there is a growing realisation that this leads to inefficient and ineffective utilisation of resources.^7,8^ Thus, there is a need for context specific evidence on the best approaches to integrate research evidence in decision-making processes that do not overlook the complex effects of inequalities.^9,10^ Accurate data are essential for health care planning, evaluating progress towards specific health targets and health care programme performance.^11,12^ Moreover, data informed decision-making is a proactive and interactive process^13^ warranting the production of quality, relevant, contextual and comprehensive data in order to improve the health outcomes.^14,15^ Hence, effective monitoring and supervision of programmes depends on the complete, accurate and timely flow of data between Primary Health Care (PHC) facilities, hospitals, District, Province and the National information hub.^16^ The first step in ensuring effective delivery of an intervention within a health care system is to provide staff and the health system managers with access to reliable, timely, contextual data that reflect the processes of care and clinical outcomes.^11,16^ However, there remains the challenge of poor utilisation of data for action, as use of data has been insufficiently integrated into decision-making processes and the information needs of decision-makers are not represented in data collection efforts.^17^

Lack of disaggregated data at municipal ward level often leads to non-evidence-informed decisions. This is because aggregated data particularly at national, provincial and district levels make it difficult to identify where the problems occur and who is responsible. The provision of aggregated data has led to failure to determine disease prevalence at a municipal ward level resulting in failure to implement targeted interventions for health improvements. This has deprived the district of an opportunity to ensure equitable distribution of resources. Moreover, information needs to be transformed into data for action. At Amajuba district PHC service delivery sites, municipal ward-based health data are consistently collected but seldom used for taking action to improve child health outcomes. This paper describes and analyses the application of municipal ward-based health data in decision-making, in order to improve child health outcomes.

## Research methods and design

### Research design

A cross-sectional, observational study using a mixed methods’ approach involving collection of both quantitative and qualitative data was used. Quantitative data were collected using a semi-structured questionnaire, and qualitative methods were used to elicit information through focus group discussions (FGDs) using an interview schedule.

### Study setting

The study was conducted in Amajuba NHI pilot district, KwaZulu-Natal Province, South Africa at 25 PHC service delivery sites situated in a hybrid of rural, urban and peri-urban areas. Dannhauser is predominantly rural with 1 ward out of 11 wards which is peri-urban. Emadlangeni is rural with farms and one ward out of four which is urban. Newcastle has 31 wards of which 23 are urban and 6 are peri-urban and 2 are rural.

### Study population and sampling strategy

The study population comprised PHC operational managers (OMs), PHC professional nurses (PNs) and ward-based outreach team (WBOT) leaders and PHC supervisors working in public PHC service delivery sites (except for those employed for less than 6 months). Employees from Dannhauser community health centre were also excluded as the centre had been operational for less than 6 months. The first 6 months were considered as an orientation period where an employee may not be well versed with the processes. These sites included fixed PHC and mobile clinics and PHC services provided by WBOTs. Random sampling was used for the quantitative aspects of the study. The sample size was determined using stratified random sampling based on the operational hours of all clinics and the number of PNs available in each. Purposive sampling was used to select FGD participants. PHC service providers were the respondents (*n* = 131), from a total finite population of 198 PNs which produced a two-sided 95% confidence interval with a precision of 5% assuming maximum variability.^18^

Names of people in the target population on duty were written on pieces of paper and put in a bowl and randomly selected until the required number per site was obtained. Purposive sampling was used to select FGD participants. There were four PHC supervisors and nine WBOTs, who were all included. The OMs were randomly selected; names were put in a bowl and selected.

### Data collection

A semi-structured questionnaire, which investigated how the collection of municipal ward-based health data influenced the decision-making process, was used to collect quantitative data. Data on the use of municipal ward-based health data to make informed decisions, provision of targeted interventions, development of child health dashboard indicators and the disease burden list were obtained.

Five FGDs were conducted. The participants of FGDs were OMs (two FGDs of five persons each); WBOTs’ leaders (two FGDs comprising 5 and 4 individuals) and PHC supervisors (one FGD with four supervisors). The total number of respondents for the FGDs was 23, comprising 22 females and 1 male who was a WBOT leader and the only available male. Two research assistants used an interview guide to facilitate the FGDs. The FGDs were audio recorded with the permission of the participants.

In addition, a document review was done using the PHC Register (facility, mobile and WBOT) and summary sheets from January to December 2015, to assess the extent to which municipal ward-based health data collection informs the decision-making process to improve child health outcomes. Triangulation of data was undertaken, comparing data from the questionnaire, FGDs and document review.

### Data analysis

All quantitative data were coded and captured in EpiData Version 7 and analysed using STATA Version 13. Continuous variables were summarised as means and standard deviations while categorical variables were summarised using frequencies. The researcher transcribed the audio recordings to familiarise herself with the data. The qualitative data analysis entailed reading, re-reading and coding the data using the NVIVO software package. Content and thematic analysis was used to understand the data.

### Measures to ensure validity and reliability of the study

The pilot was undertaken with enrolled nurses; 10 participated in the pre-test for the questionnaire and 5 of these nurses participated in the FGD pilot.

Research assistants were trained by the principal investigator on the concept of the municipal ward-based health data and the questionnaire. It was emphasised to participants that confidentiality would be maintained, allowing them to provide honest responses. Further training was done on conducting FGDs and the use of the guiding themes, which included how to pursue a theme until saturation, whilst ensuring participation by all respondents.

### Ethical considerations

Ethical approval was obtained from the University of KwaZulu-Natal Humanities and Social Sciences Research Ethics Committee (HSS/0676/015D). Informed written consent was obtained from all study participants. Written permission to access data and health facilities was granted by the Epidemiology, Health Research and Knowledge Management Unit, KwaZulu-Natal Department of Health and the Amajuba District Department of Health.

## Results

### Quantitative data

The majority of the participants were PNs who provide the clinical component for most PHC clinics. The study suggests that the years of service significantly influenced the responses (see Table 1).

**TABLE 1:** Categories of participants.

Categories of participants *N* = 131	Frequency (%)	Years employed: Mean (s.d.)	P*
1. PHC supervisor	1 (0.76)	6 (-)	< 0.001
2. Facility manager	22 (16.79)	13.7 (8.5)	
3. PHC nurse	99 (75.57)	6.6 (5.9)	
4. WBOT	9 (6.87)	5.0 (4.0)	

*Source*: Research questionnaires

s.d., standard deviation

* , years of service influenced the responses.

Table 2 summarises the quantitative data collected: 78 (60%) of the participants developed the child health indicator dashboard (focus indicators monitored constantly) and 71 (56%) developed the burden of disease list for their catchment area, informed by collected municipal based health data, yet only 59 (48%) provided targeted interventions to a large extent and 24 (19.5%) to a certain extent with even fewer (46 [36%]) using municipal ward-based health data to a large extent and 28 (22%) to a certain extent.

**TABLE 2:** Participants’ responses regarding application of municipal ward-based health data to inform decisions.

Response	Frequency (%)
**Extent of provision of targeted child interventions**	
1. Not at all	6 (4.88)
2. Limited extent	22 (17.89)
3. Not sure	12 (9.76)
4. Certain extent	24 (19.51)
5. Large extent	**59 (47.97)**
**Use of data**		
Reporting	26 (20.31)
Planning	**21 (16.41)**
Evaluation	1 (0.78)
Not used	2 (1.56)
1, 2 & 3	**54 (42.19)**
1 & 2	**24 (18.75)**
**Developed own indicators for health outcomes**	
1. Yes	**78 (60.47)**
2. No	43 (33.33)
3. Do not know	8 (6.20)
**Developed burden of disease list**		
1. Yes	**71 (55.81)**
2. No	32 (24.81)
3. Do not know	26 (19.38)
**Municipal ward data to inform decisions**	
1. Not at all	8 (6.2)
2. Limited extent	26 (20.16)
3. Not sure	21 (16.28)
4. Certain extent	**28 (21.71)**
5. Large extent	**46 (35.66)**

*Source*: Research questionnaires

Values in bold denote positive disposition to the use of municipal ward-based health data.

### Data from the focus group discussions

The study findings are presented based on two themes: The role of information in the health system and inculcating a culture of evidence informed decision-making.

#### Theme 1: The role of information

The participants understood the role of information in the health system. They viewed it as assisting in planning for resource requirements per facility, such as human, financial and material resources. Some felt it was essential for strategic planning and for planning awareness campaigns. It was evident that the role of information in planning interventions was a common thread across all five FGDs. The planning for awareness campaigns and child health week was mentioned in all FGDs. The participants also shared a common understanding of the role of information in that it informs targeted interventions as captured in the quotations below.

The respondents stated that the health system needs to know the community served, and that correct information will enable them to meet community needs. Some respondents stated that the municipal ward data helps in evaluating the child health outcomes. Other participants said that the municipal ward based data helps the system’s understanding of the local epidemiology in order to address the burden of disease and to promote community involvement. Participants generally felt that the main purpose of ward based data is to help inform interventions to be executed in a specific municipal ward. Some stated that municipal ward data helps to understand the catchment population health, informs response to disease outbreak and sharing information with relevant stakeholders. There was consensus that the contextual data informs resource allocation.

‘Help us to direct resources to the interventions we want to undertake.’ (PHC Supervisor, female, nursing, 10years at PHC level)

‘It can also help perhaps that you need some more resources, for example there I am lacking a Community Care Giver (CCG) in such and such an area and yet there is an outbreak or there is some uprooting conditions in a certain area - maybe I need a CCG in such an area. It informs your establishment what type of resources or human resources you might need in the near future.’ (OM, female, nursing, 15 at PHC level)

‘It can inform the budget, as you plan for next budget as to which areas we focus on, if you have focus areas from municipal ward based health data.’ (WBOT, female, nursing, 2 years at PHC level)

‘To give a clear picture or understanding as to what is happening in a specific area to plan for interventions. Child health week planned in response to challenges identified.’ (OM, female, nursing, 8 years at PHC level,)

‘It is used as a tool to establish the disease profile per catchment population to make us understand which diseases are more prevalent for a specific population and for us to direct the interventions accordingly as a department and we need to focus more on primary prevention strategies.’ (PHC supervisor, female, nursing, 13 years at PHC level )

‘The health system needs to know the needs of the community it is serving. It has to have the correct information from the community in order to meet these needs.’ (WBOT, male, nursing, 7 years at PHC level)

#### Theme 2: Culture of evidence informed decisions

In all focus groups participants emphasised the important role of the analysis of the data in the facility information review meetings. These meetings are attended by the relevant health care providers, although some WBOTs did not attend them. The participants believed that feedback from the district office on data collected would enhance utilisation of the information by employees at facility level and that this enables planning for targeted interventions. This feedback entails information on patients who accessed health services from a health facility outside the catchment area of a particular facility, as they have a right to access services in a facility of their choice as long as it is the correct level of care. Furthermore, importance of planning on how the community needs should be met was expressed, as some WBOTs were not members of the facility information review committee. The need to develop a quality improvement plan, and how this should be implemented and recorded in the information committee record book was emphasised. Community involvement was considered to play an important role in the dissemination of the health information and health promotion strategies in order to prevent the spread of diseases.

‘I think after data collection we need to do data analysis with responsible people and decide the way forward. Like let us say there is an outbreak of measles, malnutrition, sit down, look at data and make a plan how we are going to overcome this, intervene and report on it.’ (WBOT, female, nursing, 2 years at PHC level)

Some WBOTs stated that data collection and reporting tools should be available and that the OM should monitor their utilisation and give feedback to the staff, stating that a person acting for the OM must be familiar with what is happening in the context. From these statements it can be assumed that the OM is perceived as the knowledgeable person; it can be argued that the OM does not share information or empower subordinates, hence the perception by the WBOT. The staff stated that data should be accurately captured and reported the following morning during team briefing meeting to facilitate evidence based decisions. The PHC supervisors, OMs and some PNs said that the absence of data capturers in the facilities negatively influenced completeness and accuracy of data and thus contributed to its non-utilisation, as they are responsible for data collation from different registers. This demonstrates that not all data handlers and users appreciated each other’s contribution as all WBOTs did not mention this as a challenge. The OM emphasised the importance for the WBOTs to write reports instead of giving verbal feedback when attending to referrals, so that there would be evidence of the intervention. Moreover, whatever intervention is implemented should be recorded in order to document the responsiveness to the community needs and to confirm that the intervention was informed by municipal ward based data. Surprisingly, previously the OMs had not addressed the issue of verbal reports, although the WBOTs report to them, in order to ensure completeness of the records and evidence of the interventions implemented.

All FGDs stated the importance of reporting and using data to inform decisions, Furthermore, they agreed that there was a need for in-service education for all the PHC staff on how information influences decision-making.

‘What normally happens is that WBOT respond to referrals and give back a verbal report or no report. They will report verbally there is no evidence. Until you see them the next day and ask what did they discover. It will encourage everybody to record.’ (OM, female, nursing, 8 years at PHC level)

‘Conduct in-service education to inform staff about the value of information how it influences decision making with regard to resource allocation and service delivery.’ (PHC Supervisor, female, nursing, 6 years at PHC level)

### Record review

In some facilities, there was erratic documentation of the municipal ward in the register, but other facilities implemented the municipal ward-based health data collection accurately. Monthly summary sheets of collated data were available as was the record of the targeted interventions implemented. The evidence corresponded well with the quantitative results.

The results show that not all health workers collect the municipal ward-based health data. Those who collected data knew the purpose of collecting it. The minutes of the war room meetings (meetings attended by sector departments, civil society and municipal ward councillors to address the social ills) showed that there was a platform for discussing health needs and in particular child health, providing an opportunity for discussing which interventions needed to be undertaken. There was no standardised reporting format for targeted interventions.

It was evident that not all employees were aware of the child health dashboard indicators and the disease burden list, and yet these were available in the facilities where they were working or to which they were linked. Yet, this was necessary for understanding the context to enable planning for targeted interventions. The use of disaggregated data to inform decision-making was not embraced by all respondents. Action plans were available in some facilities, but no proof of implementation based on municipal ward based data was available. Respondents stated that they had implemented targeted interventions, but there was erratic documentation.

## Discussion

Reflecting on the responses with regard to the extent to which municipal ward-based health data were used to make data informed decisions to improve child health outcomes, indicated underutilisation, although respondents provided targeted interventions to improve child health outcomes. This contradiction indicates poor understanding and appreciation of the role of data to inform appropriate interventions. Moreover, developing child health indicators and the burden of disease list is essential to monitor and plan interventions to improve child health outcomes by using data to inform the decisions. This is supported by the literature that confirms that the use of data has not been adequately integrated into decision-making processes and that the information needs of decision-makers are often not adequately represented.^14,17,19^ The results suggest that there is a need to continue to promote application of the municipal ward-based health data in decision-making to improve child health outcomes.

The findings of the study show that the WBOTs were knowledgeable and well oriented about the health information system. There was no consistency between the level of knowledge of the role of information and its application in informing decision-making to improve child health outcomes, despite emphasis on maximising local use of data in this study. Yet, literature shows the value of local use of data as information is relevant only if it is used to resolve a local problem or when it assists in generating innovations that solve a local problem.^20,21,22^ Similar observations have been made in Malawi where the traditional thinking of collecting data for reporting purposes is deeply rooted.^20,21^ The results reveal that the majority of participants used municipal ward-based health data for planning. The results were confirmed during the FGDs in that municipal ward based data were being used for planning awareness campaigns, health education and interventions that were to be implemented. There was a challenge in developing action plans with some facilities having an action plan but no minutes indicating what interventions were implemented. It could not be assumed that because there was an intervention written in the action plan that such had been implemented, hence the need for a reporting template as proof of implementation. This will facilitate monitoring of any implementation of an intervention. The need to inculcate a culture of evidence informed decisions was identified in this study and participants identified the need for in-service education.^23^ This may be beneficial as lessons from Malawi demonstrate that the reviewing of the curriculum and their job descriptions contributed to a remarkable achievement in using information for action.^20^ Participants in this study agreed that this needs a collective effort, with monthly meetings to consider the data, and analysis of the municipal ward based data, then developing an action plan, implementing this, monitoring the implementation and recording deliverables. Evidence in the literature encourages investing in strengthening systems for data collection and management.^9,19^

The results reveal poor teamwork as some PNs from fixed clinics were neither aware of the dashboard indicators nor participated in their development. Mobile facilities and WBOT are attached to a fixed facility, and sharing of information is vital and they need to have been part of the development of dashboard indicators based on the disaggregated facility data. The tendency of blaming was observed with the OMs blaming the WBOTs for not giving written reports and the WBOTs blaming the former for not including them in the facility information review meetings. Both these categories work together, and hence this indicates that poor personal relationships are prevalent, and that there is a need to strengthen teamwork.

This study contributes to health transformation in the Amajuba district. The provision of targeted interventions informed by municipal ward-based health data will contribute towards universal access and the managing of scarce resources.^24^ There is compelling evidence indicating that informed decision-making in public health offers numerous potential benefits.^4,25^ These include adoption of the most effective and cost-effective interventions and sensible use of resources, resulting in better health outcomes for individuals and communities, hence the need for innovative approaches for health systems strengthening for ensuring sustainability.^4,25^ There is insufficient coverage of all the wards by the WBOTs thus the availability of the municipal ward based data will contribute to understanding the population health and their specific health needs. This shortage of WBOTs was stated as a concern in all FGDs in that it negatively influences the completeness of data and understanding of population health. Hence, decisions can be skewed and not address the specific needs of the community if based only on those who have accessed health services in a fixed facility. Furthermore, the poor have limited access to health services, and by not providing outreach services they may access services late, already experiencing complications. That will be expensive to manage and contribute to further depletion of the scarce resources. If evidence informed decisions are used to shape the health service delivery platform, cost effectiveness can be achieved and child health outcomes improved. This innovation can be applicable in any PHC setting and can be scalable to other districts and provinces.

Understanding the health information landscape and the movement towards e-health, this innovation (collection of municipal ward-based health data to inform decision-making) has not been implemented elsewhere. In Amajuba, the municipal ward-based data are collected at all the PHC service delivery sites. This enables understanding of the context in order to be responsive to the actual community needs, and it is also aligned to the NHI deliverables of responsiveness to community needs and universal access. The study focused at the PHC level only, excluding district, regional and the private health sector, hence generalisations are limited to the PHC level.

## Recommendations

The health service providers need to be re-oriented about the decision-making process to make evidence-informed decisions. The need for community participation and education is important in understanding the value of municipal ward-based health data and the need to give correct residential addresses which facilitate tracing of patients when necessary. Information is one of the fundamental components of the decision-making process, but currently it does not appear to be prioritised. Managers at all levels of care should advocate for the use of information for action. It is imperative to involve data users and producers when making decisions. They can collect, analyse, synthesise, interpret and use data in decision-making. In view of the rising cost of health care and the scarcity of resources, scarcity can be managed by the provision of targeted interventions. Targeted interventions can contribute to public value. Amajuba district needs to strengthen the implementation of municipal ward-based health data to enable evidence-informed decisions which can greatly contribute to improving child health outcomes.

## Conclusion

We require substantial investment in the PHC information system to produce information that can be used at a local level to influence decision-making. Without it, it is less likely that we can improve population health. The innovation has great potential in improving population health, and the support for further strengthening must continue until a culture of information use is created in the entire health system. More innovative action-oriented research is needed to develop an implementation model to integrate municipal ward-based health data into the DHIS.
